# “It's the First Barrier” – Lack of Common Language a Major Obstacle When Accessing/Providing Healthcare Services Across Europe

**DOI:** 10.3389/fsoc.2020.557563

**Published:** 2020-11-05

**Authors:** Florence Samkange-Zeeb, Silja Samerski, Lucy Doos, Rachel Humphris, Beatriz Padilla, Hannah Bradby

**Affiliations:** ^1^Department of Prevention and Evaluation, Leibniz Institute for Prevention Research and Epidemiology – BIPS, Bremen, Germany; ^2^Faculty of Social Work and Health, University of Applied Sciences Emden/Leer, Emden, Germany; ^3^Institute of Cancer and Genomic Studies, University of Birmingham, Birmingham, United Kingdom; ^4^School of Politics and International Relations, Queen Mary University of London, London, United Kingdom; ^5^Department of Sociology, University of South Florida, Tampa, FL, United States; ^6^Centre for Research and Studies in Sociology, ISCTE – Instituto Universitário de Lisboa, Lisbon, Portugal; ^7^Department of Sociology, Uppsala University, Uppsala, Sweden

**Keywords:** language, obstacle, migrants, superdiversity, healthcare

## Abstract

International migration is shaping and changing urban areas as well as impacting on healthcare access and provision in Europe. To investigate how residents of superdiverse neighborhoods put together their healthcare, we conducted qualitative interviews with 76 healthcare providers and 160 residents in four European cities - Bremen, Germany; Birmingham, UK; Lisbon, Portugal and Uppsala, Sweden, between September 2015 and April 2017. A common theme arising from the data was language and communication obstacles, with both healthcare providers and users experiencing language difficulties, despite all four countries having interpretation policies or guidelines to address language barriers in healthcare. Official interpreter services were seen to be unreliable and sometimes of poor quality, leading to a reliance on informal interpretation. Some coping strategies used by both service providers and users led to successful communication despite the lack of a common language. Where communication failed, this led to feelings of dissatisfaction and frustration among both users and providers. Language difficulties came up across all participating countries even though this was not prompted by interview questions, which highlights the widespread nature of language barriers and communication barriers and the need to address them in order to promote equal accessibility to good quality healthcare.

## Introduction

Despite the extensive literature on how the lack of a common language impairs access to quality healthcare provision (Flores, 2005; Bauer and Alegria, 2010), on-going globalized migration makes it imperative that this topic be revisited. Language skills and the ability to articulate a problem in consultations are crucial to accessing healthcare (Dixon-Woods et al., 2005). Without such skills, people are prevented from accessing the necessary services, and hence from receiving appropriate care (Ahmad and Walker, 1997).

To accommodate the language needs of different population groups, European national health services have taken several measures, including interpreter services, multi-lingual health information, training in working with interpreters and maintaining a register of staff language skills (Huddleston et al., 2015; McGarry et al., 2018). The standard measures applied in the United Kingdom (UK), Portugal, Sweden and Germany, the four countries in which the current study was conducted, are described below.

National Health Service (NHS) Trusts in the UK are obliged to ensure that ethnic minorities can understand healthcare information, and that patients and clinicians can communicate effectively. According to NHS guidance, rather than relying on family members or friends, healthcare professionals should offer a professional interpreter (Public Health England, 2014).

In Portugal, government agencies should inform immigrants and professionals of their rights and duties, as well as mediate when difficulties arise, for example when a language barrier exists between professionals and users (Equinet – European Network of Equality Bodies, 2016).

In Sweden, anyone who cannot speak Swedish adequately has the formal right to get an interpreter when seeking health and dental care (Migrationsverket, 2017). However, the availability of skilled interpreters varies greatly.

In Germany, every patient has the right to be adequately informed and advised about any procedures in a language he/she understands, yet who should cover the costs is not specified (Bundesministerium der Justiz und für Verbraucherschutz, 2013; Bühring, 2015). As in Sweden, healthcare in Germany is decentralized with different models of dealing with language barriers, even within the same federal state.

While the measures described are effective in some cases, they are insufficient in others (Greenhalgh et al., 2007; Priebe et al., 2011; Mangrio and Sjögren Forss, 2017). Moreover, the influx of refugees to Europe in 2015–2016 has further stretched healthcare systems' capacities in receiving countries, especially where refugees lack pre-existing social networks.

In this article we report on how language emerged as one of the main barriers to healthcare access and provision in our research: a qualitative content analysis of semi-structured interviews conducted with healthcare users and providers in a study conducted in four superdiverse European cities during and after the refugee crisis of 2015/16. We not only describe how language shaped the experiences of healthcare providers and users, but also describe coping strategies to suggest where service improvement is needed.

The term “superdiversity” describes the formation of new and complex social constellations in urban areas marked by the dynamic interplay of various characteristics including age, sex, educational background, country of origin, mode of migration, legal status and length of stay in area/new country (Vertovec, 2007). Such neighborhoods serve as arrival zones for new migrants, who tend to locate to areas in which people they know, or know of, reside, to access networks to support them find their way in the new country of residence, thereby reducing the stress of relocating (McKenzie and Rapoport, 2007; Zaiceva and Zimmermann, 2014).

## Study Context, Materials, and Methods

The data used were collected as part of the project Understanding the practice and developing the concept of welfare bricolage (UPWEB), whose aim was to further advance the concept of welfare bricolage in order to increase understanding of how residents of superdiverse neighborhoods in four European cities put together their healthcare (Phillimore et al., 2015). UPWEB comprised mixed methods, including a qualitative approach (semi-structured interviews, street mapping and ethnographies) and a survey based on the qualitative findings. The fieldwork was conducted in two different neighborhoods located in Bremen, Germany, Birmingham, UK, Lisbon, Portugal and Uppsala, Sweden.

UPWEB adopted a broad and innovative operationalization of who constituted a *healthcare provider*, thereby encompassing a spectrum of persons working in public or private healthcare institutions, or with organizations involved in the neighborhood offering health related services, support and mediation for healthcare access. The term includes medical personnel, social welfare officers, community or non-governmental organization (NGO) workers and mediators among others, all of whom will hereon simply be referred to as “provider.” By *residents*, we mean persons who at the time of the study were living in the study neighborhood.

A total of 236 semi-structured qualitative interviews were conducted between September 2015 and April 2017 with healthcare providers (*n* = 76), and residents (*n* = 160) of 8 superdiverse neighborhoods, as part of a wider study looking at how residents of superdiverse neighborhoods put together their healthcare. The interview questions focused on approachability and accessibility of local services for local populations and how service providers and service users overcame the barriers they encountered. Each of the participating countries has a different type of welfare state system (Rice, 2013) and the cities selected have long histories of migration, and are each home to people from more than 100 different national origins. Both neighborhoods selected in each city are characterized by superdiversity, with one of them exhibiting a high level of economic and social deprivation and the other showing signs of redevelopment (see Supplementary Table 1 for characteristics of selected neighborhoods).

The study was approved by the relevant body in each project setting: the Ethics Review Committees of the Universities of Birmingham and Bremen, the Lisbon and Tagus River Regional Health Authority of Lisbon and the Region Uppsala (Etiknämnden, diarienummer 2015/112).

The sampling technique aimed for maximum variation for selecting participants. To assist in mapping the neighborhoods, identify potential interviewees and co-conduct interviews, multi-lingual persons who were familiar with the respective neighborhoods and participated in different social activities within the community were recruited and trained as community researchers. Community researchers went through an application and selection process at each of the participating study centers and worked together with academic researchers (Phillimore et al., 2019a,b). The age, sex and languages spoken by the community researchers are provided in Supplementary Table 2.

Service providers were identified via ethnographic mapping as well as during the resident interviews. All interviewees, both residents and providers, were interviewed once, and informed about the study aims and procedure at recruitment and again before the actual interview. They were also informed that the interview would be recorded and the transcribed data pseudonymized. Participants signed informed consent forms, which were available in different languages.

Residents were asked questions regarding how they had dealt with a health concern experienced since living in the respective neighborhood: what they did, their sources of support in getting healthcare, the resources they used as well as any problems or obstacles they encountered in accessing healthcare (Phillimore et al., 2019a).

Providers were asked about the challenges they faced while conducting their work in the neighborhood, as well as the challenges faced by their patients/clients when accessing services.

In some instances the community researchers also served as interpreters during the resident interviews, and some of them also transcribed and translated transcripts of interviews conducted in a language other than the country's main language.

Key issues raised by the interviewees were identified using a systematic thematic analysis approach (Phillimore et al., 2019a,b). To this end, a common codebook was developed cooperatively between the research teams, both for the resident and the provider interviews. Throughout the inductive process, the codebooks were tested on at least two interviews in each country and comments and suggestions that were made by the coders were incorporated in their further development, with the process being moderated across the four countries.

## Results

### Description of Study Population

#### Residents

The 160 residents interviewed varied regarding age, sex, country of birth, nationality, length of stay in their respective country/neighborhood and proficiency in local language (Table 1). The proportion of those with no or basic local language competency ranged from 15.6% in Portugal, to 27.5% in Germany. In general, poor local language competency seemed to be related to short duration of stay in the country (up to 5 years) in Germany, Sweden and Portugal. In the UK however, six of the seven residents with poor local language competency had been in the country for at least 9 years.

**Table 1:** Characteristics of residents interviewed in the four participating countries.

	**Germany**	**Portugal**	**Sweden**	**UK**	**Total *n* = 160**
	**total *n* = 40**	**total *n* = 45**	**total *n* = 35**	**total *n* = 40**	**n (%)**
	**n (%)**	**n (%)**	**n (%)**	**n (%)**	
**Gender**					
Male	16 (40.0)	18 (40.0)	15 (42.9)	21 (52.5)	70 (43.8)
Female	24 (60.0)	27 (60.0)	20 (57.1)	19 (47.5)	90 (56.3)
**Age**					
18–29	3 (7.5)	7 (15.6)	7 (20.0)	11 (27.5)	28 (17.5)
30–44	19 (47.5)	19 (42.2)	7 (20.0)	10 (25.0)	55 (34.3)
45–59	11 (27.5)	11 (24.4)	9 (25.7)	8 (20.0)	39 (24.3)
60–79	5 (12.5)	6 (13.3)	7 (20.0)	10 (25.0)	28 (17.5)
80+	2 (5.0)	2 (4.4)	5 (14.3)	1 (2.5)	10 (6.3)
**Local language competency**					
None/basic	11 (27.5)	7 (15.6)	9 (25.7)	7 (17.5)	34 (21.3)
Good/very good	3 (7.5)	2 (4.4)	–	–	5 (3.1)
Fluent	12 (30.0)	6 (13.3)	12 (34.3)	33 (82.5)	63 (39.4)
Native/mother tongue	14 (35.0)	30 (66.7)	12 (34.3)	–	56 (35.0)
Missing	–	–	2 (5.7)	–	2 (1.3)
**Born in country of current residence**					
Yes	16 (40.0)	20 (44.4)	12 (34.3)	11 (27.5)	59 (36.9)
No	24 (60.0)	25 (55.6)	23 (65.7)	29 (72.5)	101 (63.1)
**Employment status**					
Working for pay/profit	18 (45.0)	22 (48.9)	17 (48.6)	21 (52.5)	78 (48.8)
Unemployed	14 (35.0)	11 (24.4)	4 (11.4)	5 (12.5)	34 (21.3)
Domestic tasks	3 (7.5)	3 (6.7)	3 (8.6)	4 (10.0)	13 (8.1)
Retired	3 (7.5)	4 (8.9)	6 (17.1)	5 (12.5)	18 (11.3)
Permanently sick	2 (5.0)	3 (6.7)	2 (5.7)	–	7 (4.4)
Student	–	1 (2.2)	3 (8.6)	3 (7.5)	7 (4.4)
Other	–	1 (2.2)	–	2 (5.0)	3 (1.9)
^*^Years living in country of current residence					
	Total *n* = 24	Total *n* = 25	Total *n* = 23	Total *n* = 29	Total *n* = 101
≤5	11 (45.8)	9 (36.0)	4 (16.7)	6 (20.7)	30 (29.7)
6–10	2 (8.3)	3 (12.0)	3 (13.0)	3 (10.3)	11 (10.9)
11–20	5 (20.8)	4 (16.0)	10 (43.5)	9 (31.0)	28 (27.7)
>20	6 (25.0)	8 (32.0)	6 (26.1)	10 (34.5)	30 (29.7)
Not collected	–	1 (4.0)	–	1 (3.4)	2 (2.0)

* *only for those born outside the country of current residence*.

#### Providers

The professional backgrounds and categories of institutions represented by the 76 providers interviewed varied within and between countries. The majority of providers interviewed were healthcare professionals, among them medical doctors, nurses, physiotherapists and pharmacists from the state and private sector, as well as civil society organizations. Other service providers such as educators, social workers and acupuncturists were however also interviewed.

**Figure 1:**
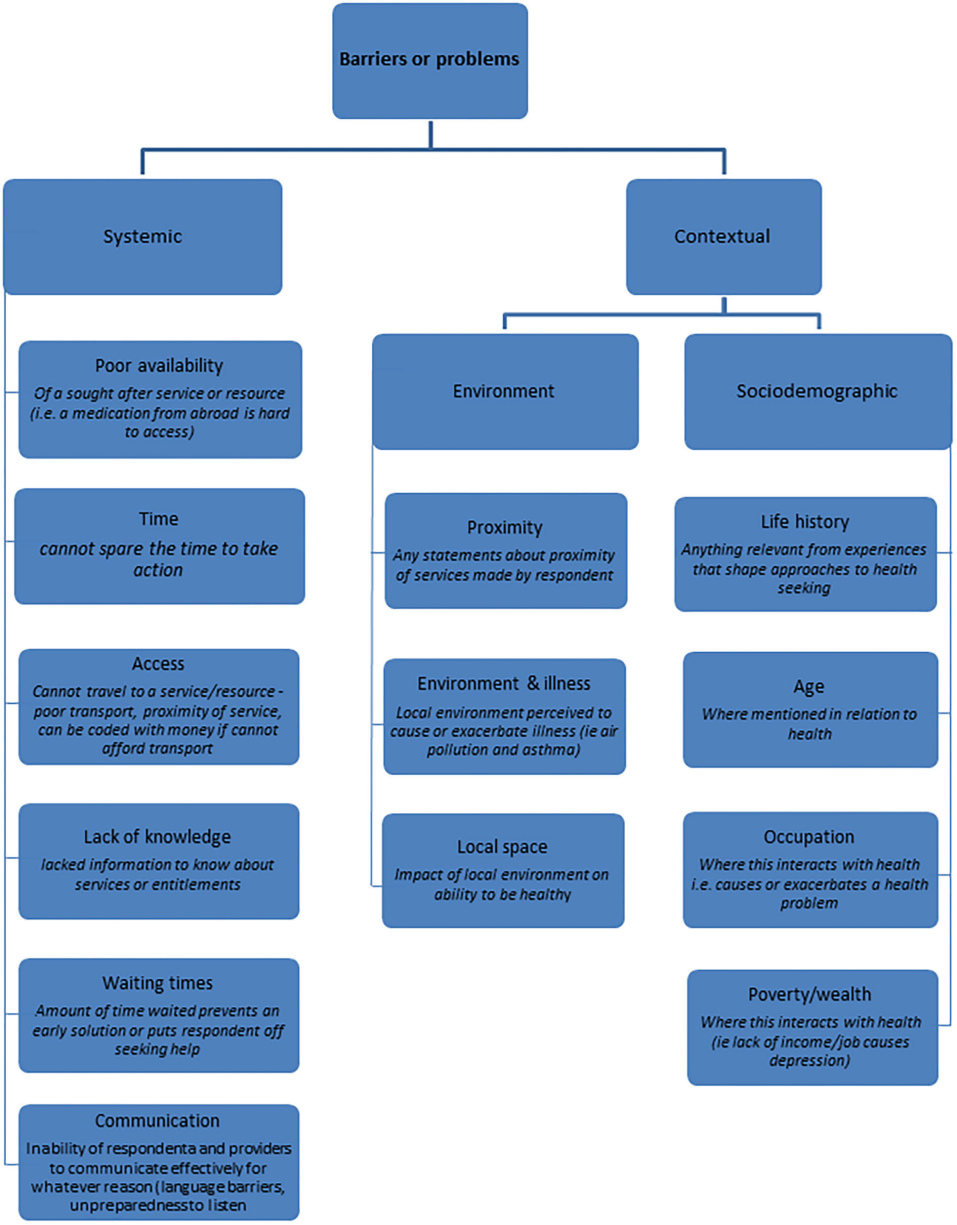
Identification of themes regarding barriers that emerged from the thematic analysis.

The main themes regarding barriers that emerged from the thematic analysis are presented in Figure 1. In addition to waiting times, lack of knowledge about existing services and poor availability of services, lack of a common language (communication) was one of the main barriers to accessing/ providing healthcare service pointed out by both residents and providers across all four countries.

### Expressed Sentiments Related to Not Being Able to Speak the Local Language Adequately

One of the main issues that arose from the interviews with residents who had a migrant background was how the lack of a common language hindered the process accessing healthcare. This affected men as well as women of all ages, regardless of level of education.

As expressed by a 29 year-old Birmingham resident (interview language Mandarin):

*The main barrier is with language. After all, English is not our language, when the issue is complicated, we're not able to understand the language, especially at a medical appointment, if you don't understand, or you're not sure about what you heard, when you miss out a message, or misunderstand something, it'll be bad*.

This feeling of not being able to speak and understand the local language sufficiently emerged across several interviews in all four countries and in some cases led to people avoiding going to the doctor altogether.

An example is the case of Tinka, a 38 year-old mother of two and originally from Bulgaria, who at the time of the interview had been in Germany for 8 months. She had lower abdominal pains and burning sensations when passing urine, but was putting off going to the doctor until she had learnt enough German to be able to communicate with the doctor directly.

Tinka had one friend she trusted to accompany her to the doctor, but said the friend could not always make time, given her own family commitments. She rejected the idea of asking anyone else to accompany her or using an interpreter, saying that her condition was too private and she finds it uncomfortable to talk about it via a third person. In the meantime, she said that she would take painkillers she gets from her home country.

Her general wish, she said, was,

“*that we don't get ill, that the children don't get ill. That we don't have a situation where we have to go to the doctor because it's so difficult as we don't speak the language.”*

A 58 year-old male from Hong Kong interviewed in the UK also identified language barriers and issues with interpreters as the most prevalent obstacle when addressing health concerns. He explained that although interpreter services are available, he doesn't really want to use them believing that his health is a private matter.

*The thing is, it's my personal issue. I don't want to expose it to another person. And, the only way is to have my wife to go with me; she is the closest person to me. Whatever I say, whatever secret, I don't mind letting her know; if it is the interpreter, I'd hesitate, not sure if I like to tell; it's very private, and not sure if it's okay to tell. I can't pass that barrier from myself*.

He added that his wife sometimes accompanies him as she speaks better English, but occasionally she mentions unnecessary details; to his dismay.

### Speechlessness, Frustration, Resignation

Feelings of speechlessness and frustration due to not being able to communicate adequately were evident not only in resident interviews, but also those with providers. In some provider interviews this also lent a tone of discrimination and racism to what was being said when patients were blamed for their inability to speak the local language.

For example, a family doctor in Portugal related:

*This happens a lot with immigrants. Often they come alone, they don't speak Portuguese and don't speak English and it's just impossible. I sometimes need to tell them to come back with someone that speaks the language… Sometimes we can't even understand the bases, how they feel, where it hurts, how it hurts, since when it hurts. it's very frustrating for the professionals and I think it is as well for the patient. It's the first barrier*.

Pria, an academic with fluent English, was dismissed by healthcare professionals in Lisbon on account of her Indian accent. She attended a hospital consultation for a high risk pregnancy and the English-speaking doctor refused to communicate in English, saying that Pria should speak Portuguese, the language of the country. Furthermore, the consultation was attended by a group of interns, to which Pria had not consented.

A gynecologist in Germany explained that the lack of communication negatively influenced the interaction with his patients: “*Where I also don't really know how I should actually cope. Because that's incredibly frustrating and hm leads to, I also then notice, that at the same time also leads to, that one becomes aggressive.”*

Feeling discriminated against because of an inability to speak the local language was mentioned by several residents and was confirmed by some providers. A midwife in one of the UK neighborhoods commented:

*I think if you don't speak English you suffer all sorts of kind of like indirect racism and prejudice and that affects the care that you have. You see it on the wards*.

### Availability and/or Use of Formal Interpreter Services

Some providers in the UK, Sweden and Portugal mentioned having used, tried to use, or considered using interpreter services. The interpreter services that are supposed to be available were, however, reported to be difficult to access, the booking procedure was tricky and at times challenging, while on many other occasions, situations were unexpected, so could not be planned ahead. According to some providers, even when interpretation services could be accessed, they did not necessarily improve the communication and sometimes introduced new communication problems.

A supervisor of a mental health organization in the UK described a session with an interpreter she had witnessed:

*It had the effect of the client not understanding the material. Because I speak that language, so I knew that person is not communicating the information effectively to the client. I was just observing, as a trainee, so I have observed quite a few. So I wasn't very happy with how she communicated back to the client*.

Frequent reference to the poor and variable quality or non-availability of interpreters at healthcare encounters was also mentioned in the interviews with residents and healthcare providers in Sweden. For instance, an elderly Iranian explained that she was satisfied with her healthcare but “*dissatisfied with the translations provided by an interpreter,”* and a healthcare screening worker for new arrivals confirmed that there are “*few interpreters*” and “*even if the interpreter appears, everyone does not interpret well*.”

In some cases, residents reported that they were not offered services of an interpreter, despite this being legislated. In Sweden, an Arabic-speaking 50-year old woman described how her husband or daughter usually accompanied her to physiotherapy sessions to treat a painful shoulder muscle. When her family could not accompany her, she had to make do with her own extremely limited Swedish-language, since no interpretation was made available. Another interviewee, originally from Palestine, described receiving overnight hospital emergency care after a traffic accident before having learned Swedish, but being offered no interpreter.

In Portugal, most of the providers interviewed said they did not make use of the existing interpreter services which they described as being inefficient or inadequate, as the existing translation system requires phone interface and in advance scheduling. They instead relied on volunteers who accompany the patient, tried using a third common language such as English or French, or even gestures.

In the UK and Germany it was mentioned that even if interpreters are requested, they were not necessarily available when needed, leading to the reliance on informal interpreters such as family members or friends. Further, in Germany it is unclear who is supposed to pay for the interpreter services, leading to some providers avoiding such services and instead leaving it to the patient to ensure that he/she brings someone to help out. None of the residents in Germany mentioned having made use of or being offered formal interpreter services.

### Self-Help Strategies

Both healthcare providers and residents described a range of strategies in the face of language barriers. Making use of in-house staff and volunteers who are multi-lingual was a common strategy used by providers in all four countries. For example, a pharmacist in Germany described how a Turkish-speaking colleague was shared between the pharmacy and two doctors operating from the same premises.

Whereas, some providers said it is up to the patient to bring someone who speaks the local language along, others go out of their way to help their patients/clients, as exemplified by a pharmacist interviewed in Portugal who said:

*Yes, yes when I have a doubt, or a sick person who isn't Portuguese and who has troubles in expressing, I prefer to contact the doctors directly to be sure. Sometimes I do it by phone, sometimes by letter. It depends on the situation*.

Another strategy mentioned by providers in Germany and proved to be effective was the use of gestures, “mit Hand und Fuss,” as they generally call it.

Residents generally mentioned taking relatives or friends along or calling them on the phone to ask for interpretation and some health professionals mentioned using Apps or Google translate. In Germany, two residents mentioned that they had approached strangers who spoke their language on the street and asked them to accompany them to the doctor's and had had to pay for the services.

### Language and Trust

Both providers and residents pointed out the importance of building relationships between provider and patient/user, and how this is reflected in sharing a common language. Using interpreters and multilingual volunteers was said to help build trust and improve access to the service provided.

A mental health supervisor in the UK stated:

*It is about being able to speak the same language as them. It makes them feel comfortable and it builds that relationship of trust in that short space of time that you have and you just feel that connection with them because you speak their language and they open up to you*.

A German female resident of Turkish background, who at times accompanies elderly Turkish people to the doctor as an interpreter, commented:

*Really, if doctors can only speak three, four Turkish words, whether it's hallo or bye, they already have plus points. Then the people are friendlier, more open. And if he then offers him the hand in greeting and says hallo in Turkish, a load falls off, because he thinks he'll be understood. And ahm, there are many problems with people who don't speak German, they are lost at the doctor's*.

### The Role of the English Language in Bridging the Gap

In Germany those without English competency reported more problems communicating, whereas patients who spoke English fared very well even if they didn't speak German, as almost all providers could speak and understand English. In fact, one of the interviewees went as far as to say that he expected all doctors to be able to speak his language (English), and that he would not go to anyone who could not speak English. In Portugal, as noted, some health professionals discriminated against patients who spoke English with a non-European accent, claiming that this was not English.

## Discussion

Although the study from which this analysis is drawn did not focus on language barriers and sampled for the range of groups in the neighborhood (not just immigrants/minorities), the issue of language, communication and interpretation, in relation to trust, came through as a key issue across our interviews. Language barriers were the most common challenge mentioned by residents with a migrant background and also by providers working with this population group. For non-migrants and for others who could speak the local language confidently, it was nonetheless important that the service providers did not “treat me like a number.” Even when using the same language as their healthcare providers, residents sometimes felt deeply frustrated and disappointed with the care that they received (Bradby et al., 2020).

The language barriers observed in our study not only contributed to residents delaying or avoiding going to the doctor, but also to their being handled brusquely and unfairly. The negative experiences recounted by the residents indicate the need for more work to be done in the health sector to combat discrimination, prejudice and racism, and enhance intercultural communication skills among medical personnel as well as empathy toward patients.

The complexity of using family members as medical interpreters is well-depicted in our study, where although one interviewee preferred to have his wife interpret for him, saying his health was a private issue which he would not want to discuss through a stranger, he is however frustrated by the extra information that she includes in describing his case to the healthcare professional.

Given that formal commitments have been made to provide interpretation services in healthcare settings in Portugal, Sweden, Germany and the UK, the level of unmet needs and frustration uncovered by this study provides evidence not only of the serious nature of these gaps, but also their ubiquity. In addition to reiterating the difficulties faced when trying to incorporate formal interpreters during the normal course of care provision (Mangrio and Sjögren Forss, 2017; McGarry et al., 2018), the study also points toward a lack of acceptance of some of the measures put in place to combat language barriers on the part of providers, such as the use of telephone interpreters. This indicates that care providers and users/patients need to be consulted when measures are being drawn up.

Developments taking place in technology such as the use of video interpreting services can perhaps fill the need for qualified interpreters who are readily available for routine healthcare encounters. Video services are anticipated to be better than telephone services as they allow visual communication and are more similar to face-to-face interpretation (Masland et al., 2010). This however requires that health facilities have access to high quality internet services and equipment. Further, the extent to which the issue of trust plays a role here remains unclear.

In our study, community researchers played a pivotal role in building trust with poor local language competency. As they were familiar with the cultural background of the respective interviewees, they did not merely interpret, but were co-producers of knowledge, actively mediating between the academic researchers and the interviewees (Padilla and Rodrigues, 2017; Samerski, 2020).

To conclude, while there are examples of successful healthcare communication despite the lack of a common language in all four research settings, there are instances where communication failed, putting the health of immigrants and ethnic minorities at risk. Our study shows that further action to address gaps in communication has to be taken as a matter of social citizenship, equity, and ethical and social responsibility. Since migration is unlikely to abate, attending to linguistic interpretation as an integral part of good healthcare provision is crucial and should be brought to the political agenda immediately.

## Data Availability Statement

The datasets presented in this article are not readily available because the informed consent signed by the participants did not include their agreeing to their qualitative data being shared publicly. Requests to access the datasets should be directed to Jenny Phillimore, j.a.phillimore@bham.ac.uk.

## Ethics Statement

The studies involving human participants were reviewed and approved by Ethical Review Committee of the University of Birmingham, Ethics Committee University of Bremen, ISCTE-IUL Ethical Review Committee (University of Lisbon) and Swedish Ethical Committee (Etiknämnden) in Uppsala, (diarienummer 2015/112). The patients/participants provided their written informed consent to participate in this study.

## Author Contributions

FS-Z, SS, LD, RH, BP, and HB were involved in the data collection, transcription, coding, and analysis. FS-Z drafted the manuscript. All authors revised it critically and approved the final version.

## Conflict of Interest

The authors declare that the research was conducted in the absence of any commercial or financial relationships that could be construed as a potential conflict of interest.
