# Supplementary material for: “It's the First Barrier” – Lack of Common Language a Major Obstacle When Accessing/Providing Healthcare Services Across Europe
Source: Front Sociol. 2020 Nov 5;5:557563. doi: 10.3389/fsoc.2020.557563 (PMC8022480; doi:10.3389/fsoc.2020.557563)
Supplement: Supplementary file 1 [file Table_1.docx]

**“It’s the first barrier” – Lack of common language a major obstacle when accessing/providing healthcare services across Europe**

**Florence Samkange-Zeeb^1^, Silja Samerski^2^, Lucy Doos^3^, Rachel Humphris^4^, Beatriz Padilla^5^, Hannah Bradby^6^.**

^1^ Leibniz Institute for Prevention Research and Epidemiology – BIPS, Department of Prevention and Evaluation, Bremen, Germany

^2^University of Applied Sciences Emden/Leer, Faculty of Social Work and Health, Emden, Germany

^3^University of Birmingham, Institute of Cancer and Genomic Studies, Birmingham, United Kingdom

^4^Queen Mary University of London, School of Politics and International Relations, London, United Kingdom

^5^University of South Florida, Department of Sociology, 42 E Fowler Ave, Tampa, FL, USA & Lisbon University Institute (ISCTE-IUL), Lisbon, Portugal

^6^Uppsala University, Department of Sociology, Uppsala, Sweden

*** Correspondence:**Florence Samkange-Zeeb
samkange@leibniz-bips.de

Keywords: language, obstacle, migrants, superdiversity, healthcare

Abstract

International migration is shaping and changing urban areas as well as impacting on healthcare access and provision. To investigate how residents of superdiverse neighborhoods put together their healthcare, we conducted qualitative interviews with 76 healthcare providers and 160 residents in four European cities - Bremen, Germany; Birmingham, UK; Lisbon, Portugal and Uppsala, Sweden, between September 2015 and April 2017.

A common theme arising from the data was language and communication obstacles, with both healthcare providers and users experiencing language difficulties, despite all four countries having interpretation policies or guidelines to address language barriers in healthcare. Official interpreter services were said to be unreliable and sometimes of poor quality, leading to a reliance on informal interpretation. Some coping strategies used by both service providers and users led to successful communication despite the lack of a common language. Where communication failed, this led to feelings of dissatisfaction and frustration among both users and providers.

The fact that language difficulties came up across all participating countries even though this was not the main focus of the project highlights the widespread nature of language barriers and the need to address them in order to promote equal accessibility to good quality healthcare.

**Table 1: Characteristics of the comparison countries and neighbourhoods***

|  | Health and welfare regimes | City and neighbourhoods |
| --- | --- | --- |
| Germany | Conservative welfare regime  Universal, corporatist health care system, decentralized and self-governing. Compulsory health insurance based on income covers 85% of the population. Direct access to services with choice of provider. Migrants receive a health insurance card allowing access to medical help for acute illness, pain and pregnancy. Without insurance, people must pay or use volunteer doctors, CSOs and welfare organizations. There is no functioning interpretation system. The healthcare ecosystem is very complex so people struggle to understand entitlements. The ecosystem has been transformed into a competitive health market with statutory health insurers behaving as competing corporations. Medical professionals are supposed to report irregular migrants to immigration authorities. | Bremen: 10^th^ largest city  554646 residents, 30% people from migrant background (PMB), (deprived and skilled) from 162 countries.  Gröpelingen: 35,055 residents, 44.1 % PMB, 2^nd^ highest number welfare dependants (33.3 %), high deprivation. Long history migration. Increasing welfare dependency.  Neustadt: 43,699 residents, 26 % PMB, students, migrants and middle-class. Decreasing welfare dependency with early gentrification. Long history migration. |
| Portugal | Southern European welfare regime  Health system comprised of multiple sectors including a universal national health service (NHS) with co-payment scheme and exemptions for certain populations. Health subsystems include health insurance for public servants, a growing private insurance health sector and the lottery funded charity-led parallel health service of Santa Casa da Misericordia (SCML) for vulnerable populations. The economic crisis affected provision and quality of health services as TROIKA imposed severe. Most irregular migrants’ exemptions were removed making access problematic. NHS professionals cannot report irregular migrants to authorities due to professional ethics. | Lisbon: capital & largest city  547733 residents, housing migrants from 172 countries, recent arrival of refugees.  Lumiar: 25,000 residents, 15 % migrants, high welfare dependency, high deprivation.  Mouraria: 15,000 residents, migrants from 30 countries since the 1970s. Welfare dependency paired with gentrification. |
| Sweden | Social Democratic welfare regime  Comprehensive universal system. Equity is prioritised through redistributive policies in the form of statutory and municipal taxes, benefits and services aimed at mitigating the damaging effects of poverty. The system of fiscal and non-fiscal universal benefits, distributed with little means-testing imply extensive public-sector employment in health and social care. Healthcare and welfare available to whole population for a small fee. Only immigrants with legal rights of domicile can access non-urgent care. Very limited private sector. Provision through for-profit corporations increasing. Limited austerity since Sweden’s major financial crisis and contraction of the welfare state occurred in the 1990s. Emphasis on individual responsibility, healthy living and active lifestyles. | Uppsala: 4th largest city.  202625 residents, people from migrant background from 174 countries (deprived and skilled).  Gottsunda: 9,924 residents, 53 % PMB, high welfare dependency. Long history migration. Significant municipal investment addressing social problems.  Sävja: 5,330 residents, 39 % PMB, pockets of deprivation and affluence. Few municipal resources.  Occasional social unrest. |
| UK | Liberal welfare regime  The UK's NHS introduced as a universal system with primary and secondary healthcare free to all. The past 20 years have seen constant attempts at restructuring to slow down spiraling costs. Shortages of doctors and nurses with the system said to be in crisis and Government refusing to increase the budget. Restructuring in 2013 introduced service commissioning to introduce competition, reduce costs and offer choice for health “consumers”. Widespread concerns about capacity to meet rising demand, the exacerbation of recruitment difficulties, reduced investment, long-term under-funding of mental health provision and cuts in public health and social care budgets. Immigration legislation denies undocumented migrants and failed asylum seekers free access beyond emergency care. NHS workers are expected to report and refuse to treat undocumented migrants. | Birmingham: 2^nd^ largest city.  1073045 residents, 22% foreign born, 47% ethnic minorities from 187 countries.  Lozells and East Handsworth: 31,074 residents, 44.9 % FB, 89.2 % EM, 5^th^ most deprived ward. Long history migration with recent increases and diversification.  Edgbaston: 24,426 residents, 29.2 % FB, 42.2 % EM, 34^th^ most deprived ward. More recent history migration. |

Data for Germany: 2012 national census and Arbeitnehmerkammer: Bericht zur sozialen Lage 2013

Data for Portugal: migrant definition: foreign born and ethnic minorities

Data for Sweden: foreign born and ethnic minorities

Data for the UK: 2011 Census

*Source: Phillimore et al., 2015 and 2019a

Table 2: Demographic characteristics of the community researchers who participated in UPWEB

| Sex | Age | Languages spoken | Duration of stay in neighbourhood/country |
| --- | --- | --- | --- |
| **Germany** | | | |
| F | 32 | German, English, French, Nouchi, Pidgin, Sango | 5 years/7 years |
| M | 37 | German, Arabic, English, Swedish | 2 years/13 years |
| F | 31 | German, Bulgarian, Turkish | 11 years/12 years |
| F | 34 | German, Russian | 6 years/6 years |
| F | 57 | German, Polish | 20 years/20 years |
| F | 60 | German, Arabic, English | Didn’t live in either neighbourhood, but had extensive contacts to Arabic community in one of the neighbourhoods/42 years |
| F | 31 | German, Turkish, English | 31 years/31 years |
| **UK** | | | |
| F | 45 | English, Cantonese, Mandarin | Didn’t live there, but worked as a Manager of CSO in neighbourhood / 45 years |
| F | 28 | English, French, Urdu, Punjabi, Hindi. Basic knowledge of French, Russian, Spanish | 28 years / 28 years |
| F | 44 | Turkish / Kurdish, English | Lived in adjoining neighbourhood; extensive contacts in each neighbourhood through work / 15 years |
| F | 70 | English, Hindi. Limited French, Punjabi and Urdu. | 70 years / 70 years |
| **Sweden** | | | |
| F | 45 | Somali, Swahili, Swedish, English | 20 years / 25 years |
| M | 24 | Arabic, English | 2 years / 2 years |
| F | 23 | Finnish, English, Swedish | Lived near neighbourhood / 3 years |
| F | 35 | Arabic, English, Swedish | 5 years / 18 years |
| **Portugal** | | | |
| M | 49 | Portuguese, English, Gujarati, Hindi | 34 years/ 34 years |
| F | 24 | Luso-descendant, Portuguese, English, French, German, Italian, Luxembourgish, | 2 years/ 2 years |
| F | 30 | Portuguese, some Creole | Did not live in neighbourhood, but worked in the neighbourhood in NGO for several years/ NA* |
| F | 23 | English, Nepalese, some Hindi | 1 year/ 1 year (knew the new Nepalese migrant community well) |
| M | 32 | Portuguese, English | Did not live in neighbourhood, but reached out to locals and migrants from Brazil and former Portuguese colonies in Africa /7 years |
| F | 26 | Portuguese, English | Did not live in neighbourhood , but worked in local NGO so knew residents who were migrants and autochthonous/3 years |
| F | 30 | English, Portuguese, Hindi, Punjabi | Lived in another neighbourhood but knew residents from South East Asia /NA* |

NK*: Information not available
